# A comparison of diffusion tensor imaging tractography approaches to identify the Frontal Aslant Tract in neurosurgical patients

**DOI:** 10.3389/fnins.2025.1543032

**Published:** 2025-04-28

**Authors:** Sara Kierońska-Siwak, Patryk Filipiak, Magdalena Jabłońska, Paweł Sokal

**Affiliations:** ^1^Department of Neurosurgery, Functional and Stereotactic Neurosurgery, Collegium Medicum, Nicolaus Copernicus University, Bydgoszcz, Poland; ^2^Department of Neurosurgery and Neurology, Jan Biziel University Hospital No 2, Collegium Medicum, Nicolaus Copernicus University, Bydgoszcz, Poland; ^3^Center for Advanced Imaging Innovation and Research (CAI^2^R), NYU Langone Health, New York, NY, United States; ^4^Doctoral School of Medical and Health Sciences, Collegium Medicum, Nicolaus Copernicus University, Bydgoszcz, Poland

**Keywords:** DTI, FAT, tractography, Frontal Aslant Tract, DWI

## Abstract

**Introduction:**

This study aims to present various tractography methods for delineating the Frontal Aslant Tract (FAT) and to quantify morphological features of FAT based on diffusion tensor imaging.

**Methods:**

The study includes 68 patients, for which FAT was reconstructed using the Region Of Interest (ROI)-based approach. The ROIs were defined in either SFG – Superior Frontal Gyrus (ROI 1), or SMA—Supplementary Motor Area (ROI 2). The respective endpoints were located in the Inferior Frontal Gyrus (IFG)—either in pars opercularis or in pars triangularis. For each patient, FAT was delineated using four combinations of the above ROI–endpoint pairs.

**Results:**

The highest streamline counts and fiber volumes of FAT were obtained using ROI 1 (i.e., SFG) with the endpoint in IFG pars opercularis. All subjects expressed left dominance of the pathway quantified by the higher streamline counts and fiber volumes regardless of gender. Additionally, higher Mean Diffusivity (MD) and lower Fractional Anisotropy (FA) values were observed in patients above 55 years of age than in younger patients.

**Discussion:**

FAT is a neural pathway that can be tracked based on various anatomical landmarks. Clinically, it appears that delineating FAT between SFG and the pars opercularis region of IFG is optimal, as it is directly associated with the highest number of fibers and the greatest volume of the tract contained between these points.

## Introduction

The Frontal Aslant Tract is a short association neural pathway in the brain, first described by Catani. It directly connects the superior and inferior frontal gyri, having a fraction of fibers projecting to the SMA (Catani et al., 2013). Advances in neuroimaging of brain pathways have allowed for increasingly accurate visualization of FAT and direct correlation of the pathway’s anatomy with its function (Burkhardt et al., 2021; La Corte et al., 2021; Kierońska-Siwak et al., 2024). This study aims to present various tractography methods for delineating FAT and to quantify morphological features of FAT based on Diffusion Tensor Imaging (DTI).

Visualization of FAT with tractography is challenging due to several anatomical confounds. Indeed, FAT is a small and subtle structure located in proximity to major neural pathways, such as the corticospinal or fronto-striatal tracts, which tend to overlap with FAT in tractographic images (Keser et al., 2020; Burkhardt et al., 2021; Landers et al., 2022; Kierońska-Siwak et al., 2024). Moreover, FAT is a relatively newly discovered neural pathway whose precise anatomical location and trajectory are not yet fully identified.

The role of FAT is primarily associated with verbal fluency, sentence formation, and working memory (Varriano et al., 2018; Keser et al., 2020; Nakajima et al., 2021). Damage to FAT is manifested by deficits in speech output and difficulties in recognizing the meaning of homophonic words, e.g., right, light, pipe. These symptoms stem directly from the function and anatomy of FAT. Most fibers in this pathway project to the SFG, and it is precisely the frontal lobes that largely play a role in semantic processing which involves words comprehension (Keser et al., 2020). Moreover, FAT participates in memory and attentional focus which enables recognition and differentiation of the meaning of words. In a recent study, damage to FAT has been linked with compromised executive functioning (Landers et al., 2022).

La Corte et al. (2021) presented several aspects of FAT functionality. A properly functioning FAT contributes to verbal fluency, sentence production, and lexical decision-making. It also plays a role in visuo-motor integration, as well as the planning and coordination of movement sequences. Furthermore, FAT is involved in maintaining and processing information in working memory, particularly in linguistic and spatial tasks (La Corte et al., 2021).

The authors of the study also emphasized that structural changes in the FAT are associated with various disorders, such as stuttering, ADHD, autism spectrum disorder, and post-stroke aphasia (La Corte et al., 2021).

FAT, beyond its typical linguistic functions, plays a crucial role in paralinguistic and extralinguistic functions. Burkhardt et al. described FAT as supporting executive functions, which interact closely with language tasks, such as attention, working memory, and the ability to regulate complex verbal behaviors. Furthermore, FAT is linked to visual and spatial short-term memory. Additionally, this neural pathway supports the coordination of hand movements, particularly in tasks requiring precise motor control (Burkhardt et al., 2021).

Accurate visualization of FAT can be crucial for neurosurgery. In patients with a frontal lobe tumor without neurological impairments, tractography-based reconstruction of FAT helps avoid damaging the neural pathway which otherwise would cause postoperative deficits like speech disorders (Kieronska and Słoniewski, 2020; Salvati et al., 2021; Kierońska-Siwak et al., 2024). Particularly, Baker et al. described a phenomenon occurring in cases of brain tumors directly infiltrating SMA or pre-SMA, referred to as ‘crossed FAT,’ which is associated with a higher incidence of mutism-type disorders (Baker et al., 2018).

Simultaneously, Thiebaut de Schotten et al. (2012) showed the functional relevance of FAT in disconnection syndromes, which further emphasizes the clinical importance of accurate tract delineation in clinical practice.

Currently, there are no definite guidelines for visualizing FAT with tractography. Also, the gender-dependent features of the pathway’s anatomy aren’t fully described. In this study, we demonstrate an anatomically constrained procedure for tracking FAT. For this, we consider four variants of ROI–endpoint pairs, and we compare the lengths, volumes, and quantities of streamlines with respect to genders (Shekari and Nozari, 2023).

This study aims to address these challenges by presenting a comprehensive analysis of tractography methods for delineating FAT. By comparing different ROI-endpoint combinations, we seek to determine the optimal strategy for accurate and reproducible visualization of this pathway. Additionally, we aim to quantify morphological characteristics of FAT, such as fiber counts, tract volumes, and diffusion metrics, and explore their variations across hemispheres, genders, and age groups. These findings are intended to provide a robust framework for both research and clinical applications, ultimately contributing to improved patient outcomes.

## Materials and methods

Patients qualified for the study were those with intracranial lesions not located in the vicinity of the FAT. Patients whose lesions interfered with the ROIs or endpoints were excluded.

The majority were patients with brain tumors in the occipital region, infratentorial tumors, or brainstem tumors. Some patients subsequently underwent tumor resection or biopsy procedures, while others were disqualified from surgical treatment. All patients whose brain tumors were located in the region of the FAT were excluded from the study.

Neurosurgical treatment was performed within a maximum of 14 days after MRI data collection and FAT mapping. None of the patients exhibited speech disorders of the motor or sensory aphasia type. Intraoperatively, FAT stimulation was not performed on any patient. Patients under 18 years of age and over 90 years of age were excluded from the study.

Informed consent was obtained from all participants and the study protocol was approved by the local Ethics Committee. The study was approved by the Ethics Committee of KB 532/2020.

### MRI Acquisition

All the patients were imaged at 3.0 T (Philips Ingenia, manufactured in 2015) using a 32-channel head coil. T1, T2, flair and T1 sequences after contrast administration were used for image analysis. The axial DWI sequence, T1 and T2 was performed with the following parameters: imaging; scan mode: MS; scan technique: SE; acquisition mode: cartesian; fast imaging mode: EPI; EPI factor: 45; shot mode: single-shot; diffusion mode: DTI; gradient overpuls: no; directional resolution: medium (15); number of b-factors: 2: b1-0, b2-800; echoes: 1; TE/TR: shortest: 85/3232 [ms]; slice thickness: 2.5 [mm]; slice gap: 0 [mm]; number of signal averages (NSA): 2; phase encoding: AP; FOV: 224 (FH) × 224 mm (AP) × 140 mm (RL); acquisition matrix: 92 × 90; reconstruction matrix: 128; acquisition voxel size: 2.43 mm (RL), 2.49 mm (AP), 2.50 mm (FH); recon voxel size: 1.75 mm (RL), 1.75 mm (AP), 2.50 mm (FH).

## DWI preprocessing

The images were processed in DSI Studio (Yeh et al., 2013). Image analysis in all patients was performed according to the schedule: convert dicom to nifty, import the preprocessed diffusion-weighted imaging (DWI) data, verify image quality, check for motion artifacts, and correct any distortions, aligns diffusion images to correct for subject movement and scanner-induced distortions, brain masking, reconstruction of diffusion data, ROI-based tracking, network analysis and export data.

We executed FSL’s eddy to remove motion artifacts (Andersson and Sotiropoulos, 2016) and reconstructed fiber directions using Generalized Q-Sampling Imaging (GQI) along with a deterministic tractography algorithm (Yeh et al., 2013) with the following settings: The ROIs and endpoints for tracking were selected from the ICBM152 adult brain anatomical atlas (Mazziotta et al., 2001) embedded in the software.

As the stopping criteria, the angular threshold was set at 60 degrees, the FA threshold was determined automatically by the software, and the number of generated streamlines was limited to 20,000. We applied 2 iterations of topology-informed pruning to remove false connections and set the autotrack tolerance at 24.0 mm. When reconstructing FAT, we obtained the following tract statistics: the number of tracks, the mean length, and the volume of FAT (Yeh et al., 2010, 2019).

**Figure 1:**
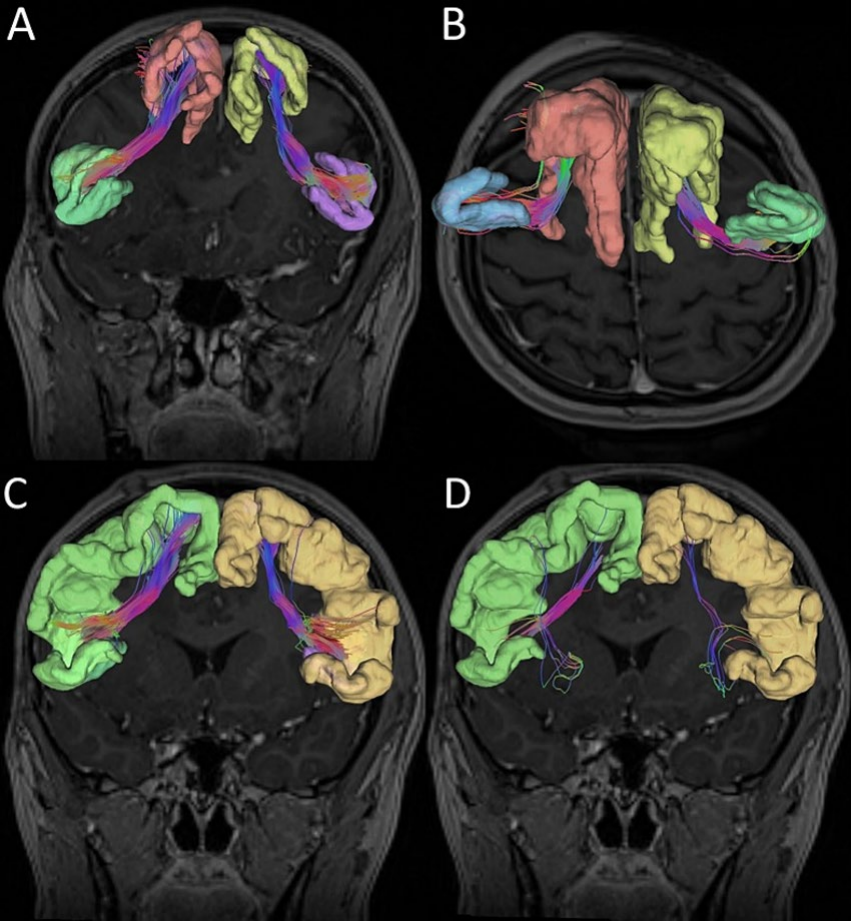
Graphic reconstruction of the Frontal Aslant Tract (FAT). **(A)** Superior frontal gyrus (ROI 1) and inferior frontal gyrus pars opercularis. **(B)** Superior frontal gyrus (ROI 1) and inferior frontal gyrus pars triangularis. **(C)** Supplementary motor area (ROI 2) and inferior frontal gyrus pars opercularis. **(D)** Supplementary motor area (ROI 2) and inferior frontal gyrus pars triangularis.

**Figure 2:**
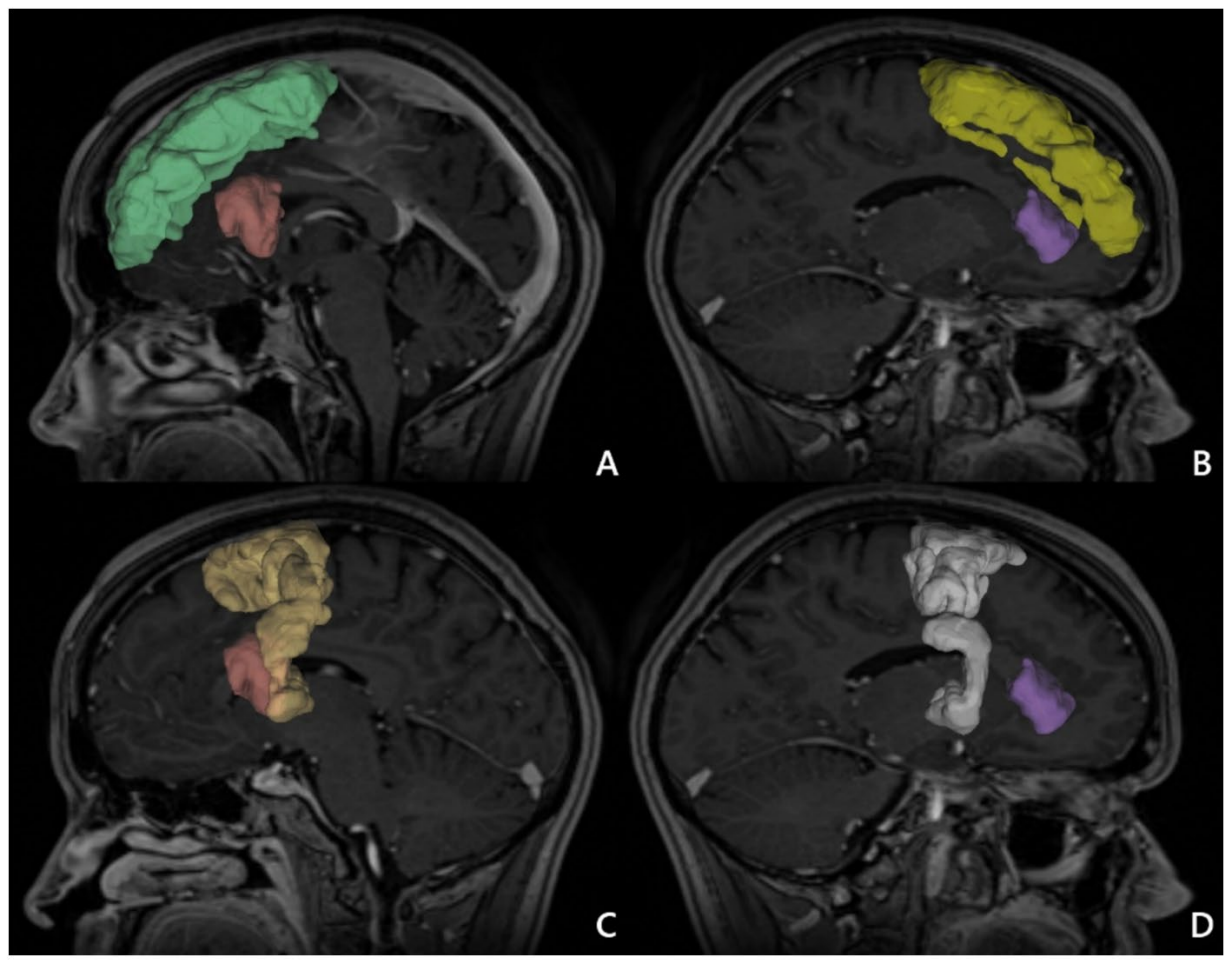
ROI in MNI brain template. **(A)** Superior frontal gyrus (ROI 1) right (green), inferior frontal gyrus pars opercularis right (red). **(B)** Superior frontal gyrus (ROI 1) left (yellow) and inferior frontal gyrus pars triangularis left (purple). **(C)** Supplementary motor area (ROI 2) right (light yellow), and inferior frontal gyrus pars opercularis right (red). **(D)** Supplementary motor area (ROI 2) left (white) and inferior frontal gyrus pars triangularis left (purple) 1.

We considered two alternative ROIs: ROI 1 – located in SFG, or ROI 2 – in SMA. Simultaneously, we defined two alternative endpoints located in the IFG—either in pars opercularis (IFG-op) or in pars triangularis (IFG-tr). For each patient, we visualized FAT in four different ways using all combinations of ROI–endpoint pairs. Next, we computed FA and MD indices along the reconstructed tracts as illustrated in Figure 1. Moreover ROI without tracts in MNI brain template was presented in Figure 2.

FAT tractography was successfully mapped for all 68 patients, with an automatic anatomical atlas used to define the ROIs (Regions of Interest). In 11 patients who exhibited supratentorial ventricular system enlargement, expansion of pericerebral spaces, or increased sizes of basal cisterns, the automatically determined ROIs were associated with incorrect anatomical placement. Consequently, manual corrections of ROI points were performed for these patients to optimize the FAT mapping method.

For data analysis in the DSI Studio software, the following parameters were set: the minimum fiber length was 30.0 mm, the maximum fiber length was 200.0 mm, the step size was 0.5 mm. These values were identical for all analyzed patients.

Topology-informed pruning (TIP) method was applied to this dataset to remove false connections. TIP algorithm can was used to automatically clean-up noisy fibers in deterministic tractography in our study, with a potential to confirm the existence of a fiber connection. TIP was applied automatically for each patient (Yeh et al., 2013, 2018). The visual inspection was conducted to identify and remove any residual false-positive streamline.

### Statistical analyses

The analysis was conducted using Statistica 13 software. The normality of the distribution of qualitative data was assessed using the Shapiro–Wilk test. In the case of conformity with a normal distribution, parametric tests were employed (Student’s *t*-test for dependent and independent variables, as well as Pearson’s rank correlation test). If the data distribution deviated from normality, non-parametric tests were applied (group comparisons using the Mann–Whitney test and, for dependent variables, the Wilcoxon test; correlation analysis utilized the Spearman rank correlation test). All analyses were considered significant at *p* < 0.001.

Additionally, statistical analysis using the Python program (Statsmodels package) and the ANOVA method were included. Due to the repeatability of measurements in all patients, mixed regression models (Mixed Effects Linear Models) were used with random effects for patients and fixed effects for tractography method, gender and age. The Paired *T*-test was used to assess hemispheric asymmetry.

## Result

We considered 68 adult neurosurgical patients (36 males and 32 females) with an average age of 53.5 years (range: 25–82 years; standard deviation [SD] = 14.69). Handedness was determined by a questionnaire completed by patients (right/left = 57/11).

FAT was delineated using four ROI–endpoint pairs. For each variant, we summarized the average values of the number of fibers, tract volumes, and lengths of tracts in all genders, obtained for the left hemisphere (Table 1) and the right hemisphere (Table 2). Notably, the highest streamline counts and fiber volumes of FAT were obtained using the combination of ROI 1 (placed in SFG) with the endpoint in IFG-op. Conversely, the lowest fiber counts and volumes of FAT were obtained for ROI 2 (SMA) with the endpoint in IFG-tr. These results were consistent in both men and women in both hemispheres (Tables 1, 2).

**Table 1:** Comparison of FAT parameters between women and men left hemisphere.

	Number of fibers (women)		Number of fibers (men)		*p* value	Volume of tracts (women) [mm3]		Volume of tracts (men) [mm3]		*p* value	Length of fibers (women)		Length of fibers (men)		*p* value
	x̄	SD	x̄	SD		x̄	SD	x̄	SD		x̄	SD	x̄	SD	
SFG-IFGop	1548.5	397.1	2231.7	119.5	**<0.001**	40806.7	9284.5	52831.7	2,399	**<0.001**	83.9	10.8	85.0	5.7	0.507
SFG-IFG triang	470.1	106.8	804.6	143.1	**<0.001**	6288.5	3790.3	8510.2	1040.9	0.203	89.7	90.9	91.4	3.9	0.243
SMA-IFGop	237.9	114.3	446.1	59.2	**<0.001**	2738.3	1374.7	5028.3	1238.1	**<0.001**	81.8	6.4	86.5	5.7	0.045
SMA-IFG triang	24.8	11	40.9	7	**<0.001**	464.8	92.5	632.3	89.6	**<0.001**	86.2	6.5	87.6	6.1	0.836

SFG, superior frontal gyrus; IFGop, inferior frontal gyrus pars opercularis; IFGtriang, inferior frontal gyrus pars triangularis; SMA, supplementary motor area; SD, standard deviation; x̄, sample mean. Bold values indicate statistically significant differences (*p* < 0.001).

**Table 2:** Comparison of FAT parameters between women and men right hemisphere.

	Number of fibers (women)		Number of fibers (men)		*p* value	Volume of tracts (women) [mm3]		Volume of tracts (men) [mm3]		*p* value	Length of fibers (women)		Length of fibers (men)		*p* value
	x̄	SD	x̄	SD		x̄	SD	x̄	SD		x̄	SD	x̄	SD	
SFG-IFGop	1422.3	397.1	2231.7	119.5	**<0.001**	37707.7	9284.5	52831.7	2,399	**<0.001**	81.5	10.5	87.5	5.4	0.492
SFG-IFG triang	411.3	106.8	804.6	143.1	**<0.001**	5987.5	3790.3	8510.2	1040.9	0.203	87.9	3.7	90.1	3.6	0.225
SMA-IFGop	216.7	114.3	446.1	59.2	**<0.001**	2533.4	1374.7	5028.3	1238.1	**<0.001**	85.6	6.1	87.6	5.2	0.038
SMA-IFG triang	22.6	11	40.9	7	**<0.001**	431.9	92.5	632.3	89.6	**<0.001**	85.4	6.3	84.3	5.7	0.792

SFG, superior frontal gyrus; IFGop, inferior frontal gyrus pars opercularis; IFGtriang, inferior frontal gyrus pars triangularis; SMA, supplementary motor area; SD, standard deviation; x̄, sample mean. Bold values indicate statistically significant differences (*p* < 0.001).

**Figure 3:**
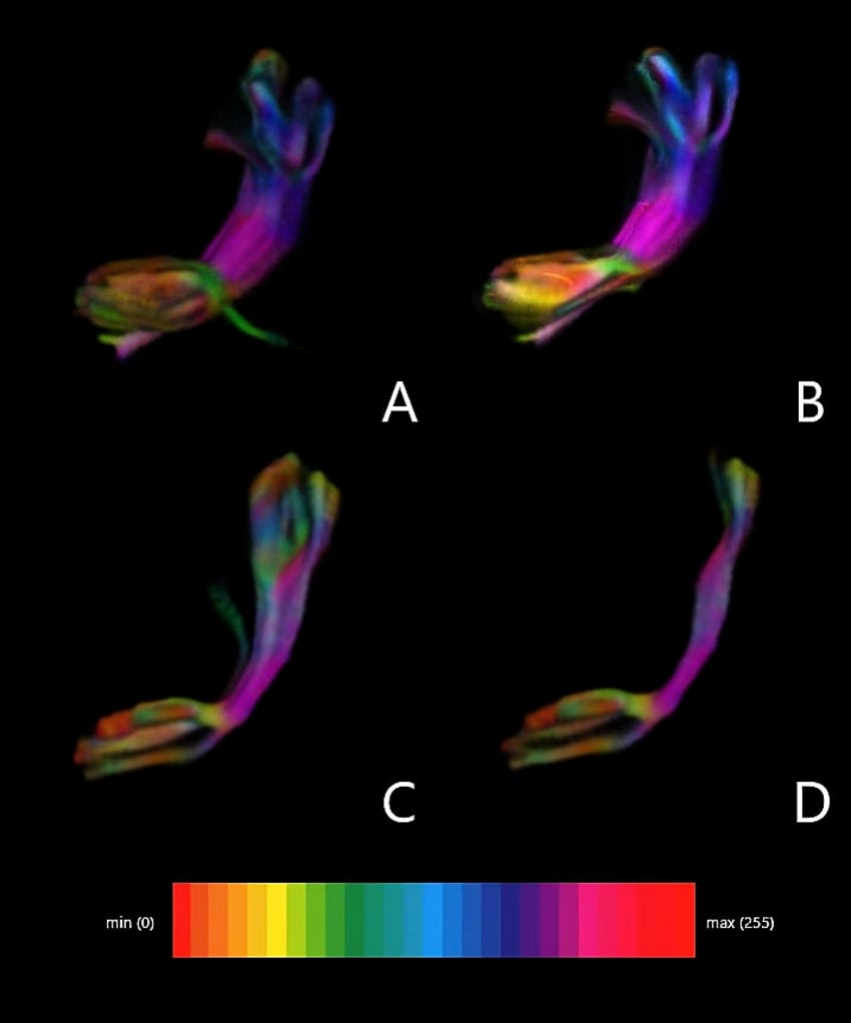
FAT density map depending on ROI. **(A)** Superior frontal gyrus (ROI 1) and inferior frontal gyrus pars opercularis. **(B)** Superior frontal gyrus (ROI 1) and inferior frontal gyrus pars triangularis. **(C)** Supplementary motor area (ROI 2) and inferior frontal gyrus pars opercularis. **(D)** Supplementary motor area (ROI 2) and inferior frontal gyrus pars triangularis.

Additionally, Figure 3 presents the FAT density map depending on the ROI.

The reconstructed FATs demonstrated a consistent interhemispheric asymmetry. Regardless of gender, the tracts obtained in the left hemisphere had significantly greater streamline counts and pathway volumes than the respective tracts in the right hemisphere. However, women exhibited lower fiber counts and pathway volumes than men, likely due to the visibly lower female skull sizes and the total volumes of the brains.

Differences in MD and FA with respect to the hemispheres and the ages of the patients are presented in Table 3. Note that FA exhibited moderately higher values in the left hemisphere compared to the right one. Simultaneously, MD was lower in the left hemisphere compared to the right one, both in men and women. However, significant differences were found when analyzing FA and MD based on the patients’ age. In the group of patients above 55 years old, FA was significantly lower, while MD was significantly higher than in the younger patients regardless of their genders. Table 4 shows differences in the number of fibers and volume of FAT without division by gender.

**Table 3:** Comparison of FA and MD values depending on the brain hemisphere and patients’ age.

	Right hemisphere	Left hemisphere	*p* value	Patient’s age 50≥	Patient’s age 50<	*p* value
FA	0.79	0.86	>0.05	0.61	0.78	**<0.001**
MD	0.78	0.72	>0.05	0.74	0.74	**<0.001**

FA, fractional anisotropy; MD, mean diffusivity. Bold values indicate statistically significant differences (*p* < 0.001).

**Table 4:** Comparison of the number of fibers and volume of FAT without division by gender.

	Number of fibers	*p* value	Volume of tracts [mm^3^]	*p* value
	x̄		x̄	
SFG-IFGop	1890.1	**<0.001**	46819.2	**<0.001**
SFG-IFG triang	637.4	**<0.001**	7399.4	0.279
SMA-IFGop	342.0	**<0.001**	3883.3	**<0.001**
SMA-IFG triang	32.9	0.509	548.55	0.305

SFG, superior frontal gyrus; IFGop, inferior frontal gyrus pars opercularis; IFGtriang, inferior frontal gyrus pars triangularis; SMA, supplementary motor area; x̄, sample mean. Bold values indicate statistically significant differences (*p* < 0.001).

Differences between tractography methods: analysis using a mixed regression model showed significant differences in FA values between different tractography methods:

SFG-IFG tra vs. SFG-IFG op: *p* = 0.005.
SMA-IF op vs. SFG-IFG op: *p* < 0.001.
SMA-IF tra vs. SFG-IFG op: *p* < 0.001.

Left–Right Asymmetry: The paired test showed significant asymmetry between hemispheres (*p* = 0.0056), suggesting greater FA values in the left hemisphere.

Comparisons between genders: There were no significant differences in FA values between men and women (*p* = 0.1704).

Among pathway lengths, no statistically significant differences were observed when comparing the left and right hemispheres, genders, and the ages of the patients.

An ANOVA test was used for FA in relation to sex and correlation with head size. There were no statistically significant differences in FA in both sexes. However, due to differences in head size in both sexes, an additional correlation was performed in this parameter. A larger head in men was not statistically significantly associated with a higher FA value.

Considering the shape of FAT, no significant differences were observed between the hemispheres. The directions of the fibers were similar, i.e., slanting from the anterior upper part of SFG to the posterior lower area of IFG.

The parameters axial diffusivity (AD) and radial diffusivity (RD) were also analyzed between the applied methods of FAT delineation. In the RD analysis, no statistically significant differences were found between the compared ROIs in FAT delineation. However, a statistically significant difference was observed in AD between SFG-IFG op (*p* = 0.035) and SMA-IF tra (*p* = 0.029). No statistical differences were found in other ROI combinations.

## Discussion

This paper emphasizes the importance of accurate delineation of FAT in the clinical context and adds to the growing evidence of the functional relevance of FAT, as detailed below.

### Tractography guidelines

In the literature so far, there is a lack of consensus about optimal tracking of FAT. Catani et al. identified certain anatomical landmarks that are helpful for delineating FAT. These include the precentral gyrus—located in the anterior part of the frontal lobe—which is associated with motor movements, and IFG—situated in the lower part of the frontal lobe—which is linked to language functions, particularly fluent speech and expression control (Catani et al., 2002; Catani and Thiebaut de Schotten, 2008). FAT typically passes through these structures, thus posing them as potentially beneficial ROIs for tracking this pathway (Catani and Thiebaut de Schotten, 2008; Dick et al., 2014, 2019).

Catena Baudo et al. (2023) presented an extension of the FAT anatomy by introducing six distinct segments of this neural pathway: superior frontal fibers connect the supplementary motor area (SMA) in the superior frontal gyrus of both hemispheres, middle frontal fibers run along the superior longitudinal fascicle (SLF), integrating motor and cognitive functions, inferior frontal fibers are crucial for speech fluency and motor planning related to articulation, insular fibers playing a role in emotional and paralinguistic processing. Cingulate fibers related to attention, working memory, and emotion processing and striatal fibers participate in planning and programming movements as well as motor control. Such a division of the FAT into distinct segments can be utilized in future analyses due to the differentiation of functions for individual parts of the FAT based on neurological disorders in patients (Catena Baudo et al., 2023).

In a similar context, Jeurissen et al. (2013) suggested alternative anatomical landmarks: the anterior cingulate cortex—located in the anterior part of the cingulate gyrus—which is associated with behavior regulation and executive functions, and the frontal pole – situated in the anterior part of the brain, just above the orbit—which is associated with executive functions, planning, and anticipation (Jeurissen et al., 2013).

Complementary to the above studies, we aimed to provide guidelines for efficient FAT delineation based on clinically feasible diffusion MRI tractography. From this perspective, we found it optimal to place an ROI in SFG and an endpoint in IFG-op. By using these specific ROIs in combination with deterministic tractography, clinicians can obtain an accurate and detailed representation of the tract (Campo et al., 2017). Our claim is supported by significantly higher numbers of fibers and volumes of the visualized tracts with respect to the other ROI–endpoint pairs considered in this paper. Analogously, Catani et al. demonstrated that the use of these ROIs results in a higher yield of fiber tract reconstructions, enhancing the overall quality of the tractography (Catani and Thiebaut de Schotten, 2008; Catani et al., 2012, 2013).

#### Fiber tract integrity

MD and FA serve as predominant metrics for evaluating white matter integrity (de Lange et al., 2016; Shekari and Nozari, 2023). Formally, MD quantifies the overall diffusion of water molecules inside a voxel of an image, whereas FA characterizes directionality of this diffusion. Note that FA ranges from 0 to 1, where 0 means isotropic diffusion (i.e., water molecules diffuse freely in all directions), while 1 means maximally anisotropic diffusion (i.e., water molecules diffuse in one preferred direction). In white matter, where diffusion occurs primarily along the axons, disrupted tissue integrity manifests as heightened MD and/or diminished FA (Bennett et al., 2010; Figley et al., 2022; Kaļva et al., 2023).

The decreased FA values that we observed along FATs in patients aged 50+ conform with the earlier reports. In this age group, white matter degradation was attributed to widening of perivascular spaces (Salat et al., 2005; Chad et al., 2018). Furthermore, changes in myelin structure and increased intercellular space affect the diffusion of water molecules and the density of nerve fibers (Salat et al., 2005; Beaulieu, 2009; Kieronska and Słoniewski, 2020; Kierońska et al., 2020).

In parallel with the decreased FA, the values of MD increased in patients over 50 years of age. Typically, this is associated with degeneration of white matter and enhancement of the extracellular space (Liu et al., 2017). Additionally, in older age, changes in arterial structure and reduced elasticity are observed, leading to decreased cerebral blood flow and reduced access to oxygen and nutrients for the brain. These changes may also affect water diffusion in the brain and contribute to increased MD (Blinkouskaya et al., 2021; Matijevic and Ryan, 2021; Ouyang et al., 2021).

### Fiber tract lengths and volumes

The lengths and volumes of FAT often bear clinical significance. Indeed, the length of FAT affects the speed of neural transmission between the regions connected by the tract (Catani and Thiebaut de Schotten, 2008; Catani et al., 2012). Note that efficient communication between SMA and the lateral prefrontal cortex is essential for coordinating speech and motor activities (Hertrich et al., 2021). Any abnormalities in the FAT length could potentially slow down signal transmission, impacting these functions. Moreover, the volume of FAT often correlates with its functional capacity (Kierońska-Siwak et al., 2024). A greater volume typically indicates a higher density of nerve fibers, which can enhance the tract’s ability to transmit multiple neural signals simultaneously. This is particularly relevant for complex tasks involving speech and motor control. Variations in the number of fibers or the tract volume can also reflect neuroplasticity, which is the brain’s ability to reorganize itself by forming new neural connections. In patients recovering from strokes or other brain injuries, changes in the FAT’s volume could indicate neurological adaptations that facilitate recovery of speech or motor functions (Catani et al., 2012; Thiebaut de Schotten et al., 2012).

The larger volumes and fiber counts of the FAT in the left hemisphere compared to the right are a result of the lateralization of language functions and the dominant role of the left hemisphere in speech and language processing. FAT is crucial for functions such as verbal fluency, lexical selection, syntactic planning, and the initiation and control of speech, which are strongly associated with the left hemisphere in most individuals, especially right-handed people (Dick et al., 2014; Burkhardt et al., 2021; Gatto et al., 2024a).

The left hemisphere FAT is a key component of the language network that connects the supplementary motor area (SMA) and pre-SMA to the inferior frontal gyrus, including the pars opercularis and pars triangularis, which enables precise control over speech processes. This structure supports both the motor aspects of speech, such as articulation and coordination of maxillofacial movements, and the cognitive components of language, including word selection and syntactic planning (Kinoshita et al., 2012).

Additionally, the asymmetry of the FAT reflects the greater functional load placed on the left hemisphere in the context of processing complex language tasks. An example of this is the role of FAT in verbal fluency, where fibers in the left hemisphere support the initiation and continuation of utterances, providing smooth transitions between words and grammatical structures (Kinoshita et al., 2012; Mandelli et al., 2014).

Neuroimaging studies also suggest that the development of the left hemisphere FAT may be a result of neural plasticity, where intensive use of language functions leads to an increase in the number of fibers and a larger volume of the structure (Pasquini et al., 2022). Furthermore, the left-sided asymmetry of the FAT is consistent with other white matter tracts that support language functions, such as the arcuate fasciculus and inferior longitudinal fasciculus (Gatto et al., 2024a).

Higher FA in the left hemisphere of FAT reflects its key role in language functions, intensive use of these structures, their better microstructural organization, and adaptive changes related to neuronal plasticity. This is a result of both lateralization of language functions and differences in white matter development and structure between the hemispheres (Dick et al., 2014, 2019; Gatto et al., 2024a).

## Clinical implications

Accurate visualization of FAT is crucial for neurosurgical interventions, particularly in patients with frontal lobe tumors. Damage to FAT during surgery can result in postoperative deficits such as speech disorders and executive dysfunction (Landers et al., 2022). Our findings underscore the importance of identifying optimal ROI-endpoint combinations to minimize the risk of such complications. Moreover, the observed correlation between age-related changes in FAT and tract integrity highlights the need for age-specific surgical planning and rehabilitation strategies (Burkhardt et al., 2021).

The clinical implication for the patient is directly tied to the accurate delineation of the FAT. For neurosurgeons operating on a patient, it is crucial to minimize the damage to neural fibers. Permanent damage to the FAT can directly result in apraxia, reduced speech fluency, difficulty generating coherent statements, aphasia, or impairments in speech planning. The choice of an appropriate method for delineating the FAT has a direct impact on planning the surgical approach and performing the craniotomy (Chang et al., 2020). The modification of the neurosurgical approach to a given intracranial pathology during preoperative planning can be achieved through the fusion of tractography images with T1, T2 contrast-enhanced, and FLAIR images. This approach can facilitate the selection of the skin incision and the dissection of appropriate brain gyri, aiming to minimize damage to the FAT (Catani and Thiebaut de Schotten, 2008).

In patients with diagnosed brain tumors accompanied by edema or tumor infiltration of neural fibers, proper planning of the craniotomy increases the chances of minimizing damage to the FAT. Additionally, employing a method that allows for the visualization of a greater number of fibers may enhance intraoperative monitoring and FAT mapping during awake surgery, thereby reducing the risk of postoperative deficits in speech and executive functions (Leote et al., 2019; Weller et al., 2023).

Previous studies have investigated the microstructural features of FAT using DWI. Tagliaferri et al. showed that anatomically FAT originates almost exclusively from the lateral portion of the SFG.

At the same time, this observation suggests a similar functional role of the SMA and the SFG convexity. The authors presented the role of FAT in the regulation of predictive strategies in motor and sensory tasks and on the functions of the prefrontal cortex. By using a combination of neuronavigated TMS stimulation and tractography, Tagliaferri et al. demonstrated that FAT plays a key role in mediating the mutual inhibition between the superior frontal sulcus (SFG) and IFG, which plays an important role in decision-making processes (Tagliaferri et al., 2023, 2024).

In our study, we also showed that the FAT projection originating in the SFG showed greater morphological parameters such as fiber number and fiber volume than the projection originating in the SMA.

Pathologies in the FAT region result in loss of speech fluency and agrammatism. FAT itself is of great clinical importance in patients with progressive apraxia of speech. In the case of patients affected by this condition, there is an impairment in the planning or programming of movements during articulation, which results in isolated errors in speech, with these errors being omitted in written statements (Catani et al., 2012, 2013). Tractography turns out to be a helpful tool in imaging pathologies at the microstructural level in patients affected by this condition. Gatto et al. showed deviations in FA and MD parameters in FAT, AF, IFOF and ILF fibers, which indicates global disruptions of the entire neuronal network involved in speech formation. At the same time, they showed that patients with progressive apraxia of speech have lower FA values and higher MD in SMA commissural fibers. Additionally, the intensity of the described abnormalities was more significant in the case of white matter fibers located on the left side, which is consistent with the lateralization of speech. The article points to the key role of the aslant part of FAT in apraxia of speech, especially in the context of progressive apraxia of speech. Diffusion-based tractography has shown reduced FAT integrity in this condition, which correlates with the severity of apraxia symptoms (Gatto et al., 2024b).

## Limitations and future directions

This study has several limitations. First, the sample size, although adequate for statistical analyses, could be expanded to include a more diverse population. Second, the use of deterministic tractography, while clinically feasible, may have limited sensitivity in resolving crossing fibers compared to probabilistic approaches. Future research should explore the use of advanced tractography techniques to validate and expand upon our findings. Additionally, longitudinal studies investigating the relationship between FAT integrity and clinical outcomes in various neurological conditions could provide valuable insights. The values of FA and MD within the FAT may change with age. Specifically, individuals over the age of 55 tend to exhibit decreased FA and increased MD, which suggests a decline in the microstructural integrity of white matter in this region. Therefore, future studies on FAT are advised to consider the participants’ age as an important factor influencing FA and MD values. Statistical corrections for age can aid in more accurate interpretation of results and a better understanding of how aging affects the microstructure of FAT (Wang et al., 2023; Aliaga-Arias et al., 2024).

Gatto et al. demonstrated that the FAT has also been studied using more advanced diffusion methods, such as multishell neurite orientation dispersion and density imaging (NODDI) (Kamiya et al., 2020). This method could provide significant benefits for future MRI studies utilizing dMRI. NODDI allows for a more detailed analysis of brain microstructure, taking into account metrics such as neurite density (ICVF) and their orientation dispersion (ODI) (Reddy and Rathi, 2016; Chong et al., 2021). In the case of FAT, the use of NODDI enables better differentiation of microstructural changes in white matter, including areas of fiber crossing, which is a significant limitation of traditional DTI imaging. The introduction of such advanced diffusion models could expand diagnostic and prognostic capabilities in the context of pathologies associated with FAT, such as apraxia of speech or aphasic disorders (Gatto et al., 2024a).

Alternative approaches to NODDI to minimize difficulties associated with crossing fibers are to use MSMT-CSD tractography, or single-shell version of it for single-shell DWI data. MSMT-CSD (Multi-Shell Multi-Tissue Constrained Spherical Deconvolution) to a method of analyzing data from diffusion imaging (DWI), which allows for more precise separation of signals from different types of tissues and fibers in the brain.

By separating the signals into several groups that are responsible for specific tissues, e.g., cerebrospinal fluid, white matter or gray matter, it is possible to obtain a more realistic model of the network of neural connections. Moreover, in the case of the problem of crossing fibers, such a model makes it easier to distinguish directions in a specific voxel (Jeurissen et al., 2014; Connelly, 2019; Hendriks et al., 2025).

In some cases it is possible to use a simplified version of the MSMT-CSD method - Single-Shell CSD for data collected with a specific b-value. In this method, tissue types are not distinguished, but it is still possible to differentiate directions in a specific voxel. Unlike MSMT-CSD, Single-Shell CSD does not distinguish between signals from gray matter, white matter and cerebrospinal fluid. In this method, the signal most often comes from the white matter, but it allows for more accurate mapping of different fiber directions, even in cases of crossing fibers (Jeurissen et al., 2014; Hendriks et al., 2025).

## Conclusion

This study presented various methods for delineating FAT in the context of clinically feasible tractography. We proposed to place an ROI in SFG and an endpoint in the pars opercularis of IFG for optimal tracking accuracy. This approach ensured a high number of fiber tracts and a substantial tract volume, facilitating reliable and detailed tractography. This precision is crucial for both research and clinical applications, ultimately contributing to better patient outcomes.

Future work should investigate correlations between the anatomy of FAT, tractography parameters, and the clinical condition of patients. Notably, the observed decrease in FA values with respect to age is a multifactorial process mainly associated with brain degeneration including structural and functional changes.

## Data Availability

The raw data supporting the conclusions of this article will be made available by the authors, without undue reservation.

## Glossary

ADHD: Attention Deficit Hyperactivity Disorder
AP: Anterior-Posterior (direction)
DTI: Diffusion Tensor Imaging
dMRI: Diffusion Weighted Data MRI
FA: Fractional Anisotropy
FAT: Frontal Aslant Tract
FH: Foot-Head (direction)
ICVF: Intra-Cellural Volume Fraction
IFG: Inferior Frontal Gyrus
IFG-op: Inferior Frontal Gyrus in pars opercularis
IFG-tr: Inferior Frontal Gyrus in pars triangularis
MD: Mean Diffusivity
NODDI: Neurite Orientation Dispersion and Density Imaging
NSA: Number of Signal Averages
ODI: Orientation Dispersion Index
RL: Right-Left (direction)
ROI: Region Of Interest
SD: Standard Deviation
SFG: Superior Frontal Gyrus
SLF: Superior Longitudinal Fascicle
SMA: Supplementary Motor Area
TIP: Topology-Informed Pruning

## References

[ref1] Aliaga-Arias J. M. Jung J. Lavrador J. P. Rajwani K. Mirallave-Pescador A. Jones A.. (2024). Asymmetry of the frontal aslant tract and development of supplementary motor area syndrome. Cancers 16:16223739. doi: 10.3390/cancers16223739, PMID: 39594695 PMC11592341

[ref2] Andersson J. L. R. Sotiropoulos S. N. (2016). An integrated approach to correction for off-resonance effects and subject movement in diffusion MR imaging. NeuroImage 125, 1063–1078. doi: 10.1016/j.neuroimage.2015.10.019, PMID: 26481672 PMC4692656

[ref3] Baker C. M. Burks J. D. Briggs R. G. Smitherman A. D. Glenn C. A. Conner A. K.. (2018). The crossed frontal aslant tract: a possible pathway involved in the recovery of supplementary motor area syndrome. Brain Behav. 8:926. doi: 10.1002/brb3.926, PMID: 29541539 PMC5840439

[ref4] Beaulieu C. (2009). “The biological basis of diffusion anisotropy,” in Diffusion MRI: From quantitative measurement to in vivo neuroanatomy (1st ed.). eds. Johansen-Berg H. Behrens T. E. J. (Oxford, UK: Elsevier Inc.). 105–126.

[ref5] Bennett I. J. Madden D. J. Vaidya C. J. Howard D. V. Howard J. H. (2010). Age-related differences in multiple measures of white matter integrity: a diffusion tensor imaging study of healthy aging. Hum. Brain Mapp. 31, 378–390. doi: 10.1002/hbm.20872, PMID: 19662658 PMC2826569

[ref6] Blinkouskaya Y. Caçoilo A. Gollamudi T. Jalalian S. Weickenmeier J. (2021). Brain aging mechanisms with mechanical manifestations. Mech. Ageing Dev. 200:111575. doi: 10.1016/j.mad.2021.111575, PMID: 34600936 PMC8627478

[ref7] Burkhardt E. Kinoshita M. Herbet G. Herbet Guillaume G. (2021). Functional anatomy of the frontal aslant tract and surgical perspectives. J. Neurosurg. Sci. 10:390. doi: 10.23736/S0390 33870673

[ref8] Campo C. A. Hernando D. Schubert T. Bookwalter C. A. Van Pay A. J. Reeder S. B. (2017). Standardized approach for ROI-based measurements of proton density fat fraction and r2 in the liver. Am. J. Roentgenol. 209, 592–603. doi: 10.2214/AJR.17.17812, PMID: 28705058 PMC5639884

[ref9] Catani M. Dell’Acqua F. Vergani F. Malik F. Hodge H. Roy P.. (2012). Short frontal lobe connections of the human brain. Cortex 48, 273–291. doi: 10.1016/j.cortex.2011.12.001 22209688

[ref10] Catani M. Howard R. J. Pajevic S. Jones D. K. (2002). Virtual in vivo interactive dissection of white matter fasciculi in the human brain. NeuroImage 17, 77–94. doi: 10.1006/nimg.2002.1136, PMID: 12482069

[ref11] Catani M. Mesulam M. M. Jakobsen E. Malik F. Martersteck A. Wieneke C.. (2013). A novel frontal pathway underlies verbal fluency in primary progressive aphasia. Brain 136, 2619–2628. doi: 10.1093/brain/awt163, PMID: 23820597 PMC3722349

[ref12] Catani M. Thiebaut de Schotten M. (2008). A diffusion tensor imaging tractography atlas for virtual in vivo dissections. Cortex 44, 1105–1132. doi: 10.1016/j.cortex.2008.05.004, PMID: 18619589

[ref13] Catena Baudo M. Villamil F. Paolinelli P. S. Domenech N. C. Cervio A. Ferrara L. A.. (2023). Frontal aslant tract and its role in language: a journey through Tractographies and dissections. World Neurosurg. 173, e738–e747. doi: 10.1016/j.wneu.2023.02.145, PMID: 36889642

[ref14] Chad J. A. Pasternak O. Salat D. H. Chen J. J. (2018). Re-examining age-related differences in white matter microstructure with free-water corrected diffusion tensor imaging. Neurobiol. Aging 71, 161–170. doi: 10.1016/j.neurobiolaging.2018.07.018, PMID: 30145396 PMC6179151

[ref15] Chang E. F. Kurteff G. Andrews J. P. Briggs R. G. Conner A. K. Battiste J. D.. (2020). Pure apraxia of speech after resection based in the posterior middle frontal gyrus. Neurosurgery 87, E383–E389. doi: 10.1093/neuros/nyaa002, PMID: 32097489 PMC7690655

[ref16] Chong S. T. Liu X. Kao H. W. Lin C. Y. E. Hsu C. C. H. Kung Y. C.. (2021). Exploring Peritumoral neural tracts by using neurite orientation dispersion and density imaging. Front. Neurosci. 15:702353. doi: 10.3389/fnins.2021.702353, PMID: 34646116 PMC8502884

[ref17] Connelly A. (2019). Single-Shell 3-Tissue CSD (SS3T-CSD) modelling of developing HCP (dHCP) diffusion MRI data. Available at: http://www.developingconnectome.org (Accessed February 16, 2024).

[ref18] de Lange A. M. G. Bråthen A. C. S. Grydeland H. Sexton C. Johansen-Berg H. Andersson J. L. R.. (2016). White-matter integrity as a marker for cognitive plasticity in aging. Neurobiol. Aging 47, 74–82. doi: 10.1016/j.neurobiolaging.2016.07.007, PMID: 27565301 PMC5404118

[ref19] Dick A. S. Bernal B. Tremblay P. (2014). The language connectome: new pathways, new concepts. Neuroscientist 20, 453–467. doi: 10.1177/1073858413513502, PMID: 24342910

[ref20] Dick A. S. Garic D. Graziano P. Tremblay P. (2019). The frontal aslant tract (FAT) and its role in speech, language and executive function. Cortex 111, 148–163. doi: 10.1016/j.cortex.2018.10.015, PMID: 30481666 PMC6461388

[ref21] Figley C. R. Uddin M. N. Wong K. Kornelsen J. Puig J. Figley T. D. (2022). Potential pitfalls of using fractional anisotropy, axial diffusivity, and radial diffusivity as biomarkers of cerebral white matter microstructure. Front. Neurosci. 15:799576. doi: 10.3389/fnins.2021.799576, PMID: 35095400 PMC8795606

[ref22] Gatto R. G. Meade G. Duffy J. R. Clark H. M. Utianski R. L. Botha H.. (2024a). Combined assessment of progressive apraxia of speech brain microstructure by diffusion tensor imaging tractography and multishell neurite orientation dispersion and density imaging. Brain Behav. 14:e3346. doi: 10.1002/brb3.3346, PMID: 38376044 PMC10761330

[ref23] Gatto R. G. Pham N. T. T. Duffy J. R. Clark H. M. Utianski R. L. Botha H.. (2024b). Multimodal cross-examination of progressive apraxia of speech by diffusion tensor imaging-based tractography and tau-PET scans. Hum. Brain Mapp. 45:e26704. doi: 10.1002/hbm.26704, PMID: 38825988 PMC11144950

[ref24] Hendriks T. Vilanova A. Chamberland M. (2025). Implicit neural representation of multi-shell constrained spherical deconvolution for continuous modeling of diffusion MRI. Imaging Neurosci. 3:501. doi: 10.1162/imag_a_00501, PMID: 40078536 PMC11894815

[ref25] Hertrich I. Dietrich S. Blum C. Ackermann H. (2021). The role of the dorsolateral prefrontal cortex for speech and language processing. Front. Hum. Neurosci. 15:645209. doi: 10.3389/fnhum.2021.645209, PMID: 34079444 PMC8165195

[ref26] Jeurissen B. Leemans A. Tournier J. D. Jones D. K. Sijbers J. (2013). Investigating the prevalence of complex fiber configurations in white matter tissue with diffusion magnetic resonance imaging. Hum. Brain Mapp. 34, 2747–2766. doi: 10.1002/hbm.22099, PMID: 22611035 PMC6870534

[ref27] Jeurissen B. Tournier J. D. Dhollander T. Connelly A. Sijbers J. (2014). Multi-tissue constrained spherical deconvolution for improved analysis of multi-shell diffusion MRI data. NeuroImage 103, 411–426. doi: 10.1016/j.neuroimage.2014.07.061, PMID: 25109526

[ref28] Kaļva K. Zdanovskis N. Šneidere K. Kostiks A. Karelis G. Platkājis A.. (2023). Whole brain and Corpus callosum fractional anisotropy differences in patients with cognitive impairment. Diagnostics 13:679. doi: 10.3390/diagnostics13243679, PMID: 38132263 PMC10742911

[ref29] Kamiya K. Hori M. Aoki S. (2020). NODDI in clinical research. J. Neurosci. Methods 346:108908. doi: 10.1016/j.jneumeth.2020.108908, PMID: 32814118

[ref30] Keser Z. Hillis A. E. Schulz P. E. Hasan K. M. Nelson F. M. (2020). Frontal aslant tracts as correlates of lexical retrieval in MS. Neurol. Res. 42, 805–810. doi: 10.1080/01616412.2020.1781454, PMID: 32552566 PMC7429310

[ref31] Kieronska S. Słoniewski P. (2020). The usefulness and limitations of diffusion tensor imaging – a review study. Eur J Transl Clin Med 2, 43–51. doi: 10.31373/ejtcm/112437

[ref32] Kierońska S. Sokal P. Dura M. Jabłońska M. Rudaś M. Jabłońska R. (2020). Tractography-based analysis of morphological and anatomical characteristics of the uncinate fasciculus in human brains. Brain Sci. 10, 1–15. doi: 10.3390/brainsci10100709, PMID: 33036125 PMC7601025

[ref33] Kierońska-Siwak S. Jabłońska M. Sokal P. (2024). Changes in frontal aslant tract tractography in selected types of brain tumours. Neurol. Neurochir. Pol. 58, 106–111. doi: 10.5603/pjnns.98149, PMID: 38230757

[ref34] Kinoshita M. Shinohara H. Hori O. Ozaki N. Ueda F. Nakada M.. (2012). Association fibers connecting the Broca center and the lateral superior frontal gyrus: a microsurgical and tractographic anatomy. J. Neurosurg. 116, 323–330. doi: 10.3171/2011.10.JNS11434, PMID: 22077454

[ref35] La Corte E. Eldahaby D. Greco E. Aquino D. Bertolini G. Levi V.. (2021). The frontal aslant tract: a systematic review for neurosurgical applications. Front. Neurol. 12:641586. doi: 10.3389/fneur.2021.641586, PMID: 33732210 PMC7959833

[ref36] Landers M. J. F. Meesters S. P. L. van Zandvoort M. de Baene W. Rutten G. J. M. (2022). The frontal aslant tract and its role in executive functions: a quantitative tractography study in glioma patients. Brain Imaging Behav. 16, 1026–1039. doi: 10.1007/s11682-021-00581-x, PMID: 34716878 PMC9107421

[ref080] Leote J. Silva M. A. See A. P. Essayed W. I. Golby A. J. (2019). Combined brain language connectivity and intraoperative neurophysiologic techniques in awake craniotomy for eloquent-area brain tumor resection. Neurosurg. Focus. 46:E9. doi: 10.3171/2019.3.FOCUS1968

[ref38] Liu H. Yang Y. Xia Y. Zhu W. Leak R. K. Wei Z.. (2017). Aging of cerebral white matter. Ageing Res. Rev. 34, 64–76. doi: 10.1016/j.arr.2016.11.006, PMID: 27865980 PMC5250573

[ref39] Mandelli M. L. Caverzasi E. Binney R. J. Henry M. L. Lobach I. Block N.. (2014). Frontal white matter tracts sustaining speech production in primary progressive aphasia. J. Neurosci. 34, 9754–9767. doi: 10.1523/JNEUROSCI.3464-13.2014, PMID: 25031413 PMC4099550

[ref40] Matijevic S. Ryan L. (2021). Tract specificity of age effects on diffusion tensor imaging measures of white matter health. Front. Aging Neurosci. 13:628865. doi: 10.3389/fnagi.2021.628865, PMID: 33790778 PMC8006297

[ref41] Mazziotta J. Toga A. Evans A. Fox P. Lancaster J. Zilles K.. (2001). A probabilistic atlas and reference system for the human brain: international consortium for brain mapping (ICBM). Philos. Trans. R. Soc. B Biol. Sci. 356, 1293–1322. doi: 10.1098/rstb.2001.0915, PMID: 11545704 PMC1088516

[ref42] Nakajima R. Kinoshita M. Okita H. Shinohara H. Nakada M. (2021). Disconnection of posterior part of the frontal aslant tract causes acute phase motor functional deficit. Brain Cogn. 151:105752. doi: 10.1016/j.bandc.2021.105752, PMID: 33993006

[ref43] Ouyang Y. Cui D. Yuan Z. Liu Z. Jiao Q. Yin T.. (2021). Analysis of age-related white matter microstructures based on diffusion tensor imaging. Front. Aging Neurosci. 13:664911. doi: 10.3389/fnagi.2021.664911, PMID: 34262444 PMC8273390

[ref44] Pasquini L. Di Napoli A. Rossi-Espagnet M. C. Visconti E. Napolitano A. Romano A.. (2022). Understanding language reorganization with neuroimaging: how language adapts to different focal lesions and insights into clinical applications. Front. Hum. Neurosci. 16:747215. doi: 10.3389/fnhum.2022.747215, PMID: 35250510 PMC8895248

[ref45] Reddy C. P. Rathi Y. (2016). Joint multi-fiber NODDI parameter estimation and tractography using the unscented information filter. Front. Neurosci. 10:166. doi: 10.3389/fnins.2016.00166, PMID: 27147956 PMC4837399

[ref46] Salat D. H. Tuch D. S. Hevelone N. D. Fischl B. Corkin S. Rosas H. D.. (2005). Age-related changes in prefrontal white matter measured by diffusion tensor imaging. Ann. New York Acad. Sci. 1064, 37–49. doi: 10.1196/annals.1340.009, PMID: 16394146

[ref47] Salvati L. F. De Marco R. Palmieri G. Minardi M. Massara A. Pesaresi A.. (2021). The relevant role of navigated tractography in speech eloquent area glioma surgery: single center experience. Brain Sci. 11:436. doi: 10.3390/brainsci11111436, PMID: 34827434 PMC8616013

[ref48] Shekari E. Nozari N. (2023). A narrative review of the anatomy and function of the white matter tracts in language production and comprehension. Front. Hum. Neurosci. 17:1139292. doi: 10.3389/fnhum.2023.1139292, PMID: 37051488 PMC10083342

[ref49] Tagliaferri M. Amorosino G. Voltolini L. Giampiccolo D. Avesani P. Cattaneo L. (2024). A revision of the dorsal origin of the frontal aslant tract (FAT) in the superior frontal gyrus: a DWI-tractographic study. Brain Struct. Funct. 229, 987–999. doi: 10.1007/s00429-024-02778-4, PMID: 38502328

[ref50] Tagliaferri M. Giampiccolo D. Parmigiani S. Avesani P. Cattaneo L. (2023). Connectivity by the frontal aslant tract (FAT) explains local functional specialization of the superior and inferior frontal gyri in humans when choosing predictive over reactive strategies: a tractography-guided TMS study. J. Neurosci. 43, 6920–6929. doi: 10.1523/JNEUROSCI.0406-23.2023, PMID: 37657931 PMC10573747

[ref51] Thiebaut de Schotten M. Dell’Acqua F. Valabregue R. Catani M. (2012). Monkey to human comparative anatomy of the frontal lobe association tracts. Cortex 48, 82–96. doi: 10.1016/j.cortex.2011.10.001, PMID: 22088488

[ref52] Varriano F. Pascual-Diaz S. Prats-Galino A. (2018). When the FAT goes wide: right extended frontal aslant tract volume predicts performance on working memory tasks in healthy humans. PLoS One 13:e0200786. doi: 10.1371/journal.pone.0200786, PMID: 30067818 PMC6070228

[ref53] Wang D. Fan Q. Xiao X. He H. Yang Y. Li Y. (2023). Structural fingerprinting of the frontal aslant tract: predicting cognitive control capacity and obsessive-compulsive symptoms. J. Neurosci. 43, 7016–7027. doi: 10.1523/JNEUROSCI.0628-23.2023, PMID: 37696666 PMC10586535

[ref54] Weller M. Le Rhun E. Van Den Bent M. Chang S. M. Cloughesy T. F. Goldbrunner R.. (2023). Diagnosis and management of complications from the treatment of primary central nervous system tumors in adults. Neuro-Oncology 25, 1200–1224. doi: 10.1093/neuonc/noad038, PMID: 36843451 PMC10326495

[ref55] Yeh F. C. Panesar S. Barrios J. Fernandes D. Abhinav K. Meola A.. (2019). Automatic removal of false connections in diffusion MRI Tractography using topology-informed pruning (TIP). Neurotherapeutics 16, 52–58. doi: 10.1007/s13311-018-0663-y, PMID: 30218214 PMC6361061

[ref56] Yeh F.-C. Panesar S. Barrios J. Fernandes D. Abhinav K. Meola A.. (2018). Automatic Removal of False Connections in Diffusion MRI Tractography Using Topology-Informed Pruning (TIP). Neurotherapeutics 16, 52–58. doi: 10.1101/338624 PMC6361061 30218214

[ref57] Yeh F. C. Verstynen T. D. Wang Y. Fernández-Miranda J. C. Tseng W. Y. I. (2013). Deterministic diffusion fiber tracking improved by quantitative anisotropy. PLoS One 8:e80713. doi: 10.1371/journal.pone.0080713, PMID: 24348913 PMC3858183

[ref58] Yeh F. C. Wedeen V. J. Tseng W. Y. I. (2010). Generalized q-sampling imaging. IEEE Trans. Med. Imaging 29, 1626–1635. doi: 10.1109/TMI.2010.2045126, PMID: 20304721

